# The intriguing roles of Siglec family members in the tumor microenvironment

**DOI:** 10.1186/s40364-022-00369-1

**Published:** 2022-04-13

**Authors:** Kui-Ying Jiang, Li-Li Qi, Fu-Biao Kang, Ling Wang

**Affiliations:** 1 Department of Orthopedic Oncology, the Third Hospital of Hebei Medical University, Shijiazhuang, Hebei People’s Republic of China; 2 Experimental Center for Teaching of Hebei Medical University, Shijiazhuang, Hebei People’s Republic of China; 3 The Liver Disease Center of PLA, the 980Th Hospital of PLA Joint Logistics Support Force, Shijiazhuang, Hebei People’s Republic of China

**Keywords:** Siglecs, Tumor microenvironment, Immune regulation

## Abstract

Sialic acid-binding receptors are expressed on the surfaces of a variety of immune cells and have complex and diverse immunoregulatory functions in health and diseases. Recent studies have shown that Siglecs could play diverse immune and nonimmune regulatory roles in the tumor microenvironment (TME) and participate in tumor progression through various mechanisms, such as regulating tumor growth and metastasis, mediating the inflammatory response, and promoting tumor immune escape, thereby affecting the prognoses and outcomes of patients. However, depending on the cell type in which they are expressed, each Siglec member binds to corresponding ligands in the microenvironment milieu to drive diverse cell physiological and pathological processes in tumors. Therefore, we herein summarize the expression spectra and functions of the Siglec family in human diseases, particularly cancer, and highlight the possibility of therapeutic interventions targeting the TME in the future.

## Introduction

Siglec family members are specifically expressed on a variety of immune cells, including human macrophages, T cells, B cells, dendritic cells (DCs), and natural killer (NK) cells, and are often involved in many important physiological processes, including the initial activation, proliferation, and apoptosis of immune cells [1]. Siglecs play important regulatory roles in the immune response by mediating cell-to-cell or pathogen-to-cell interactions through recognition of the monosaccharide sialic acid (Sia) on the surface of tumor cells. In tumors, the glycosylation of Sia on the cell surface is likely altered, thus promoting the formation of tumor-associated carbohydrates recognized by individual Siglec members, which can transmit inhibitory signals, accelerate the progression of pathological processes and promote the immune escape of tumor cells. The Sia–Siglec axis exerts different physiological functions in humans, as it modulates the balance between self and nonself recognition and mediates cell adhesion, cell signaling, and the uptake of sialylated pathogens [2]. The binding between a carboxyl group of sialylated glycoconjugates and a Siglec molecule reduces the inflammatory response, inhibits phagocytosis and reduces cellular activation [3]. In addition, the Sia-Siglec axis is involved in the capture and presentation of antigens by antigen-presenting cells and affects the functions of antigen-presenting cells. During immune activation, Siglecs counter regulate overresponsive immune reactions upon immune stimulation by damage-associated molecular patterns (DAMPs) to aid in host immune evasion, potentially leading to cancer progression [4]. The tumor microenvironment (TME) also promotes abnormal secretion of Sia from tumor cells, which in turn stimulates the upregulation of Siglec expression in infiltrating immune cells. Siglecs can promote tumor immune escape by inducing M2-type macrophage polarization and altering the direction of T-cell differentiation and NK-cell activity. Thus, dysregulation of the Sia-Siglec axis in tumors might contribute to immunosuppressive cell signal transduction to facilitate the formation of an immune-negative microenvironment, thereby promoting tumor growth and assisting in the immune escape of tumor cells [5]. Nevertheless, some Siglec molecules can deliver activation signals to promote antitumor immune responses and enhance antitumor function in the host. In recent years, an increasing number of therapeutic agents targeting Siglecs and their ligands have been developed and used in clinical trials and represent a promising immunotherapeutic approach for tumors.

## The biology of Siglecs

**Fig. 1:**
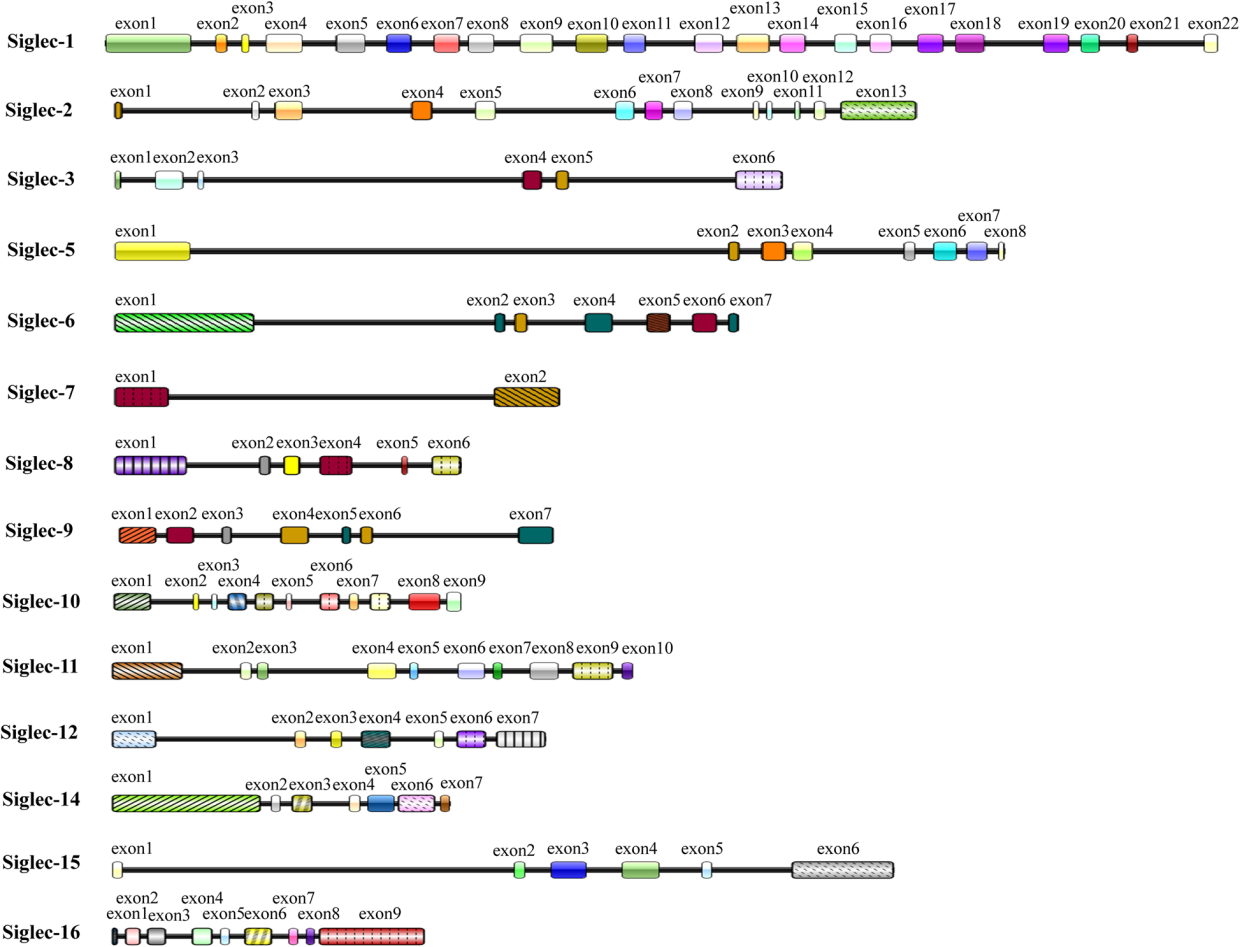
The gene structures of the Siglec family

**Fig. 2:**
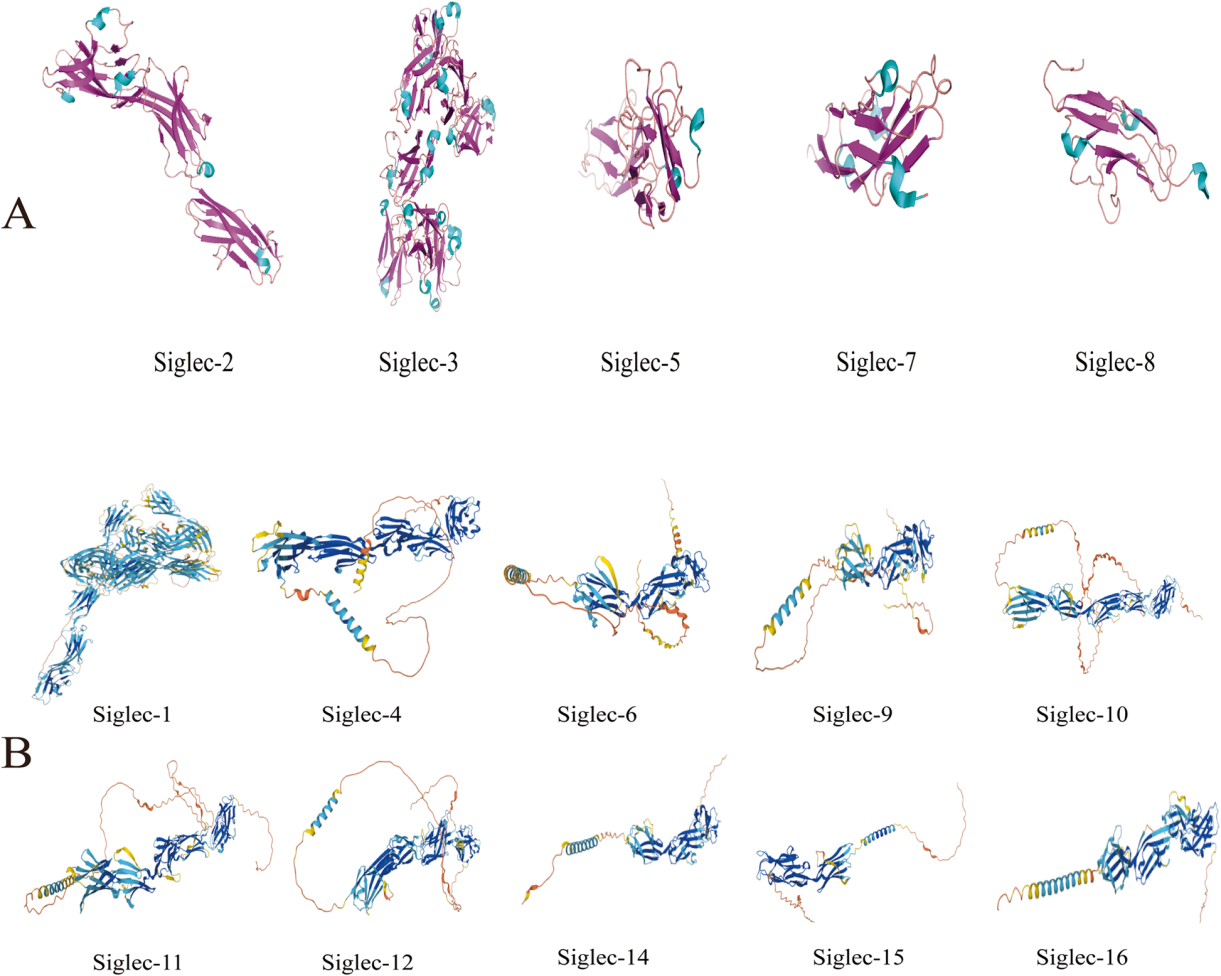
The protein structures of the Siglec family from UniProt protein database. **A** Protein structures determined using X-ray. **B** Protein structure by prediction

Siglecs are type I immunoglobulin-like transmembrane proteins consisting of an extracellular structural domain, a transmembrane structural domain, and an intracellular structural domain. The intracellular domain is divided into a short lysine-containing tail and an extracellular structural domain consisting of an N-terminal binding Ig domain and a variable number of C2-type structural domains [6]. Siglec members can exert activating or inhibitory effects depending on the specific motifs within each molecule, including the immunoreceptor tyrosine-based activation motif (ITAM) and immunoreceptor tyrosine-based inhibition motif (ITIM) [7]. Inhibitory Siglecs include Siglec-3, Siglec-5, Siglec-7, Siglec-9, and Siglec-10, and their intracellular regions contain ITIM- and ITIM-like domains, which transduce inhibitory signals by recruiting tyrosine phosphatases (SH2 domain-containing protein tyrosine phosphatases, SHPs), such as SHP-1 and SHP-2 [8, 9]. Siglecs can also be classified based on their ability to generate activated intracellular signals depending on the positively charged residue in the transmembrane region, which can interact with DAP12 carrying the ITAM domain. Human Siglec-4, Siglec-14, Siglec-15, Siglec-16 and mouse Siglec-H belong to this classification [8, 10]. Siglecs are expressed in different species of vertebrates, such as fish, amphibians, birds, reptiles, and mammals. According to the degree of sequence conservation, they can also be classified as classical conserved Siglecs and CD33-associated Siglecs. Evolutionarily conserved Siglecs, which existed in ancient vertebrates over 400 million years ago, include Siglec-1 (CD169), Siglec-2 (CD22), Siglec-4, and Siglec-15. CD33-related Siglec genes, however, developed rapidly during mammalian evolution due to multiple processes, such as gene duplication, exon loss, and gene conversion, resulting in important differences in CD33-related Siglecs among mammalian species [8]. The gene and protein structures of the Siglec family are shown in Figs. 1 and 2.

## Expression spectrum of Siglecs in tumors

The functions of Siglecs are attributed to the diverse cell types on which they are expressed, the inhibitory/stimulatory motifs they carry and the specific ligands that they bind that are involved in the interactions between tumor cells and stromal cells in the TME. Traditionally, most members of the Siglec family are expressed on major immune cell types, such as macrophages, myeloid cells, B cells and even T cells [5]. Although some Siglecs bind a set of sialic ligands with overlapping functions, they may exhibit unique specificity profiles and have differential preferences in the TME. Until now, their specific roles in the different contexts of tumors have been unclear. The expression and functions of human Siglec family members are shown in Table 1. For instance, Siglec-1 is unique to most extracellular domains among all sgRNAs and can internalize and pass antigens in macrophages or DCs. Siglec-1^+^ macrophages in the lymph nodes mostly have protective roles and are predictive of longer cancer-specific survival and better prognosis for patients with a variety of tumors, including malignant melanoma [11], colorectal cancer (CRC) [12], endometrial cancer [13], prostate cancer [14], and breast cancer [15]. However, contrasting reports have shown that Siglec-1^+^ macrophages mediate immunosuppression via JAK2/STAT3 signaling in triple-negative breast cancer cells [16], suggesting that Siglec-1 plays complicated roles in different TMEs. The Leslie Chávez-Galán team also defined TCR^+^Siglec-1^+^ macrophages as another macrophage subgroup in the TME that can potentially serve as another clinical treatment target in diseases other than cancer [17].

**Table 1:** Summary of human Siglec family members, their expression, functions, similarities and differences

Siglec	Protein Expression	Sialic Acid(SA) Ligands	Properties	Function	Disease	Refs
Siglec-1(CD169)	Macrophage,monocytes, mature dendritic cells	α2,3 > α2,6	Adhesion Cell–cell interaction	Mediates antigen presentation; inhibits the proliferation of tumor-asscociated T cell; affects TAMs' function	Cancer,autoimmunity, SLE,infectious disease	[8, 18]
Siglec-2(CD22)	B cells	α2,6	Inhibition	B cell differentiation and tolerance	Lymphoma, leukemia, SLE, sepsis, RA	[8, 19]
Siglec-3(CD33)	Myeloid progenitors, macrophage, monocytes, microglia	α2,6 > α2,3	Adhesion Inhibition	Induces apoptosis; inhibits the killing effect of NK cells; regulates myeloid cell proliferation and differentiation	AML, AD	[8]
Siglec-4(MAG)	Myelin producing cells (Oligodendrocytes, Schwann cells)	α2,3 > α2,6	Adhesion Inhibition	Adjusts axons	Neuro-degeneration	[8, 20]
Siglec-5(CD170)	Monocytes, neutrophils, B cells, activated T cells	α2,3	Inhibition	Delivers an anti-inflammatory signal; inhibits immune cell activation	GBS infection, neutrophil disorders	[8, 20]
Siglec-6(CD327)	Trophoblasts, mast cells, B cells, circulating T cells	α2,6	Inhibition	Decrease cytotoxic functions of effector CD8 + T cells; suppress trophoblast invasivenes	Cancer, pre-eclampsia, allergy	[8, 21–23]
Siglec-7(CD328)	NK cells, monocytes, mast cells, platelets, activated T cells	α2,8 > α2,6 > α2,3	Binding Inhibition	Inhibits the killing effect of NK cells; inhibits inflammatory responses of mast cells and basophils	Cancer, HIV infection, allergy	[24, 25]
Siglec-8	Eosinophils, mast cells, basophils	α2,3 > α2,6	Inhibition Apoptosis	Induces eosinophils apoptosis	Allergy, asthma	[8, 24]
Siglec-9(CD329)	NK cells, monocytes, macrophages, dendritic cells, neutrophils, activated T cells	α2,3 = α2,6, or sulfated ligands	Inhibition Apoptosis	Promotes tumor angiogenesis; inhibit the proliferation and activation of TAM,NK cells and neutrophils	Cancer, asthma, sepsis, COPD, RA	[24, 26, 27]
Siglec-10	B cells, NK cells, monocytes, CD4 + T cells	α2,3 = α2,6	Inhibition Bind to CD24	Induces apoptosis; inhibits the proliferation and activation of tumorassociated T cells and TAM	Cancer, sepsis, allergy	[27, 28]
Siglec-11	Macrophages, microglia	α2,8	Inhibition	Reduced inflammatory response; inhibits microglial activation	Neuro-inflammation, AD	[24]
Siglec-14	Monocytes, neutrophils	α2,3	Activation, Polymorphism	Recognizes bacterial pathogens, elicits pro-inflammatory responses	SLE, COPD, GBS infection,	[8, 20]
Siglec-15	Osteoclasts, macrophages	α2,6 or sialyl-Tn	Activation Inhibition	Modulation of osteoclast differentiation and bone resorption; immune modulation of tumorassociated T cells and TAM	Osteoporosis, cancer	[24, 29, 30]
Siglec-16	Macrophages	α2,8	Activation, Polymorphism	Unkown	Schizophrenia	[24]

*SLE* Systemic lupus erythematosus, *RA* Rheumatoid arthritis, *AML* Acute myelocytic leukemia, *GBS* Group B Streptococcus, *COPD* Chronic obstructive pulmoriary disease, *HIV* Human immunodeficiency virus, *AD* Alzheimer's disease

CD22 and Siglec-G are inhibitory coreceptors on the surface of B cells, and both contribute to the negative modulation of B-cell receptor (BCR) signaling by inhibiting calcium mobilization and cellular activation [19]. CD22 functions predominantly as an inhibitor in conventional B cells, while Siglec-G is an important inhibitor in the B1a cell subset. As CD22 expression is restricted to B cells, it can serve as an important immunotherapeutic target in B-cell-related lymphomas [24, 31]. Siglec-3 was found to be overexpressed on the surface of acute myelocytic leukemia (AML) cells instead of normal hematopoietic stem cells; thus, Siglec-3 can be a target for AML therapy and is being investigated in clinical trials [32]. Siglec-3 is also expressed on the surfaces of abnormal cells in patients with other myeloid disorder diseases, such as myelodysplastic syndromes, and in the B- and T-cell subsets of patients with acute lymphoblastic leukemia (ALL) [33]. Siglec-6 was recently found to be upregulated in circulating and urinary CD8^+^ T cells of patients with non–muscle-invasive bladder cancer, and high Siglec-6 expression was correlated with a low patient survival rate [21]. Siglec-6 is also expressed by mast cells in CRC tissues and may regulate the TME of CRC [22]. Additionally, Siglec-6 expression has been reported in AML blasts and B cells in subjects with chronic lymphocytic leukemia (CLL) [34] and mucosa-associated lymphoid tissue lymphoma [35]. As Siglec-6 mRNA and protein are not expressed in hematopoietic stem cells, they are novel targets for CAR-T-cell immunotherapy in CLL [20, 23].

In the TME, NK-cell-mediated cytotoxicity was shown to be strongly attenuated because Siglec-7 and Siglec-9 reactivated the interactions of NK cells with their corresponding Sia ligands overexpressed on cancer cells [9, 25]. An extensive study showed that Siglec-9 expression was detected on tumor-infiltrating T cells and that this functional effector subset of Siglec-9^+^CD8^+^ T cells was significantly inhibited in the presence of Siglec-9 ligands present on most kinds of tumor cells [36]. In ovarian and breast cancer, Siglec-10 is an inhibitory receptor expressed in tumor-associated macrophages (TAMs) that regulates immunity by interacting with CD24, suppressing immune responses, and promoting tumor progression [37].

Siglec-15 mRNA is abnormally overexpressed in most cancer types, such as breast cancer, cholangiocarcinoma, esophageal cancer, pancreatic adenocarcinoma, cutaneous melanoma, gastric adenocarcinoma, thyroid cancer, and endometrial cancer [38]. TAM-associated Siglec-15 can promote tumor immune escape by suppressing CD8 ^+^ T-cell responses and promoting immunosuppressive TME formation through the production of transforming growth factor-β (TGF-β) [29]. In addition, recent studies have shown that Siglec-15 promotes the malignant progression of osteosarcoma (OS) cells by inhibiting the DUSP1-mediated mitogen-activated protein kinase (MAPK) pathway, and high expression of Siglec-15 is associated with pulmonary metastasis and predicts poor prognosis in OS patients [39]. Li et al. suggested that Siglec-15 presented immunosuppressive relevance in pancreatic ductal adenocarcinoma (PDAC) and was expressed on TAMs and PDAC cells. Siglec-15^+^ TAMs were correlated with poor prognosis and an immunosuppressive microenvironment in the PDAC microarray cohort [40]. These results suggest that Siglec-15 has potential as a new therapeutic target.

**Fig. 3:**
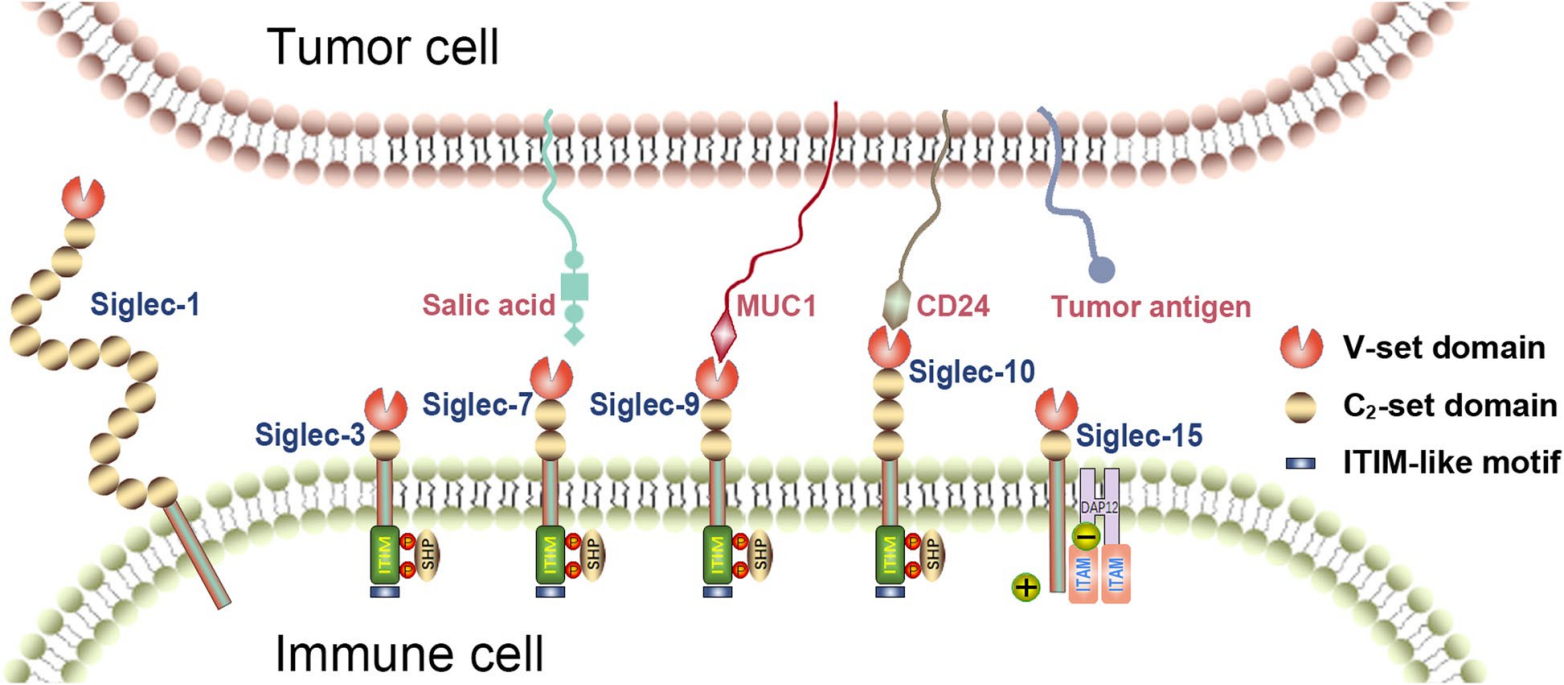
The structure schematic diagram of Siglec family members

In conclusion, Siglec family members are widely present in various tumor tissues and mostly function to negatively modulate immune responses by promoting the formation of an immunosuppressive microenvironment and tumor immune escape. A schematic of the binding between Siglec family members and their ligands is presented in Fig. 3. However, further work is required to evaluate the roles and potential mechanisms of different Siglecs on each immune cell type in the context of different cancers.

## The diverse roles of Siglec family members in tumors

### The nonimmune regulatory functions of Siglecs

#### Siglecs induce apoptosis

An important function of CD33-related Siglecs is to regulate cell growth and survival by inhibiting proliferation or inducing apoptosis [8], with Siglec-3 serving as a prominent example in tumors. Siglec-3 is mainly expressed on the surfaces of human myeloid cells and leukocytes of the myeloid lineage in AML. Chiara et al. [41] used an anti-Siglec-3 monoclonal antibody to treat AML cells and found increased DNA damage, cell cycle arrest, and apoptosis of AML cells. The mechanism was probably attributed to caveolae-dependent endocytosis and subsequent apoptosis induced by the interaction of Siglec-3 with its ligand [42]. In addition, some recently emerging monoclonal antibodies targeting Siglec-3 have been shown to bind to ligands on AML cells and directly induce AML cell death via a complement-dependent cytotoxic or antibody-dependent cell-mediated cytotoxic mechanism [43]. Moreover, Siglec-3 can promote the secretion of proinflammatory cytokines such as IL-1β, TNF-α, and IL-8 to inhibit cell proliferation.

Siglec-10 and its homolog Siglec-G are expressed in a B-cell-restricted manner, exhibiting particularly high expression levels in B1 cells, and play an important immunomodulatory role in B-cell activation [44]. Siglec-G inhibits B1-cell proliferation and Ca^2+^ signaling by suppressing the activity of the transcription factors NFATc1 and NF-kB and, upon binding to CD24, activates the MAPK-related pathway to induce the apoptosis of B cells and exert immunosuppressive effects [45]. Therefore, Siglec-10 and Siglec-G are important inhibitory receptors on B1 cells, and the lack of Siglec-G favors the development of B-cell lymphoproliferative disorders such as B-cell lymphoma/leukemia [46]. However, the mechanisms by which Siglec-10/G interact with their ligands on B cells are well understood and will be the focus of next-generation drug targets for leukemia.

#### Siglecs promote tumor angiogenesis

Siglec-9 is expressed as an inhibitory receptor on granulocytes, macrophages, NK cells, and T cells. Siglec-9 binds to MUC1 mucin on the surfaces of human colon cancer, pancreatic cancer, and breast cancer cells [47, 48], secretes tumor-related factors such as plasminogen activator inhibitor-1 [49], and promotes tumor invasion, metastasis, and neovascularization. In addition, Siglec-9 and MUC1 interact with each other to recruit and further interact with the cell cycle-associated protein β-catenin. This induces the loss of expression of costimulatory molecules and antigen-presenting molecules and phenotypic changes on the surface of Siglec-9^+^ DCs, resulting in their inability to function as antigen-presenting cells and thereby leading to the immune escape of tumor cells [50]. Moreover, Siglec-9^+^ NK cells highly express the chemokine receptors CXCR1 and CX3CR1 and can recruit IL-8 to promote tumor proliferation, invasion, and neovascularization [26]. Angiogenesis is regulated by various factors and is an important condition for tumorigenesis, development and metastasis. Therefore, the use of targeted drugs targeting angiogenic factors and their receptors will become an important strategy for the clinical treatment of tumors.

### The immune regulation of Siglecs

#### Siglecs mediate antigen presentation

Siglec-1 is mainly expressed on macrophages and plays an important role in macrophage antigen presentation [51]. Siglec-1 can help macrophages cross-present dead tumor cell antigens to CD8^+^ T cells, thereby activating cytotoxic T cells and promoting antitumor immune responses. However, blocking Siglec-1 inhibits the activity of CD8^+^ T cells in mice and affects the killing clearance of tumor cells by immune cells. Ding et al. [52] showed that Siglec-G deficiency in mice enhanced the cross-presentation of DCs by increasing the formation of MHC-I-like peptide complexes on the DC surface, resulting in enhanced cytotoxic T lymphocyte (CTL) responses and thereby facilitating the inhibition of melanoma growth. However, whether human Siglec-1^+^ macrophages can cross-present antigens and activate T-cell responses is unclear. Siglec-1 can internalize antigens and pass them on to lymphocytes by allowing DCs and macrophages to act as antigen-presenting cells, thereby making it a good therapeutic target for the development of anti-infectious and antitumor agents.

#### Siglecs inhibit the proliferation and activation of tumor-associated T cells

Siglec-1-positive macrophages were shown to be positively correlated with the number of tumor-infiltrating CD8^+^ T cells in breast cancer, which was indicative of a better response to neoadjuvant chemotherapy. Therefore, these studies suggest that Siglec-1-positive macrophages are ideal targets for enhancing antitumor immunity.

In melanoma, Quentin et al. [36] found that most tumor-infiltrating CD8^+^ T cells express Siglec-9 and that Siglec-9-mediated signaling pathways preferentially bind to SHP-1, inhibiting biological functions, including TCR signaling pathways and cytotoxicity in CD8^+^ T cells. In non-small-cell lung cancer (NSCLC) patients, Siglec-9 expression on infiltrating CD8^+^ T cells is associated with reduced survival, and its polymorphism is associated with a high risk of cancer development [53]. Siglec-10 can also inhibit TCR-mediated T-cell activation, and studies have shown that Siglec-10 expressed on the surface of T cells inhibits the phosphorylation of MHC class I molecules and the TCR-associated kinase ZAP-70, which inhibits T-cell activation [54, 55]. In addition, Siglec-10 inhibits TCR-associated kinase by binding to CD24 or CD52 to suppress T-cell activation and promote tumor immune escape [56].

Chen et al. [57] demonstrated that the upregulation of Siglec-15 expression in some tumor cells inhibited CD8^+^ T-cell proliferation and activation in vitro and in vivo in Siglec-15-deficient mice. In a mouse melanoma model, a lack of Siglec-15 promoted T-cell responses, resulting in decreased tumor growth and increased overall survival in mice. In addition, Siglec-15 promotes regulatory T-cell differentiation by inducing TGF-β, which inhibits T-cell remodeling of the immunosuppressive TME [30]. In human lung cancer tissues, Siglec-15 and PD-L1 are mutually expressed. These findings suggest that Siglec-15 plays a role in the TME and contributes to the formation of the immunosuppressive microenvironment [58]. Therefore, Siglec-15 is considered a new promising target for immune normalization independent of the PD-1/PD-L1 pathway. For patients who do not respond to PD-1/PD-L1 antibodies, targeting Siglec-15 may be an alternative. Although most T cells do not express Siglecs, they can change their glycosylation pattern and differentiate into different subtypes throughout the process of T-cell development and after activation. Siglecs not only provide inhibitory signals to their autologous cells but also inhibit the immune response to tumor cells by inhibiting the induction of regulatory T cells [59]. These results support that Siglecs function on the T-cell surface and mediate tumor immune escape; however, their role in controlling T cells and the characteristics of T cells expressing Siglecs remain to be explored.

#### Siglecs inhibit the killing effect of NK cells

Caselles et al. [60] demonstrated that Siglec-3 induces the dephosphorylation of SHP-1 molecules through the Vav1 signaling pathway and specifically antagonizes cytotoxic responses mediated by the DAP10-conjugated specifically activated receptors NKG2D and 2B4. Thus, Siglec-3 may act as an inhibitory receptor of the NKG2D/DAP10 pathway and regulate the cytotoxicity of NK cells.

Siglec-7 and Siglec-9 inhibit the NK-cell-mediated killing effect of tumor cells in vitro, and a study by Kawasaki et al. [61] found that Siglec-7 on NK cells binds to a major ganglioside (DSGb5) expressed on the surface of renal cell carcinoma (RCC) cells, thereby decreasing the cytotoxic effects of NK cells on RCC cells. In addition, Siglec-7 also interacts with a ganglioside (GD3) expressed on tumor cells and inhibits the killing activity of NK cells [62]. Thus, Siglec-7 signaling is an immune checkpoint that can be targeted to enhance the antitumor activity of NK cells [9, 63]. A recent study argued that tumor cells upregulate sialylated glycans, which counteract NK-cell-induced killing via the Siglec–sialylated glycan interaction [64]. However, the detailed mechanism remains to be explored. Some NK cells also express inhibitory Siglec-9. Jandus et al. [26] found that Siglec-9 expression was upregulated in the peripheral blood NK cells of patients with melanoma and leukemia and was mainly concentrated in the CD56^dim^CD16^+^ subpopulation, which showed lower cytotoxicity. Moreover, tumor cells showed increased expression of the Siglec-9 ligand, which reduced their sensitivity to NK-cell killing, and the killing effect of NK cells on tumor cells was significantly enhanced after Siglec-9 signaling was blocked. Enhancing NK-cell-mediated cytotoxic effects is essential for the inhibition of cancer cell survival and metastasis. Therefore, targeting Siglec-7 and Siglec-9 has emerged as a novel therapeutic approach to enhance the immune response of NK cells to cancer. The sensitivities of tumor cells with high Siglec-7 expression to the killing of NK cells in breast, brain, colon, liver and lymphoid tissues are increased after sialidase treatment. However, the Siglec-mediated modulation of NK-cell functions needs to be further explored to evaluate the potential of targeting this pathway in patients.

#### Siglecs affects TAM function

Siglec-1 has dual biological effects. In a study of hepatocellular carcinoma (HCC) in vitro, Siglec-1-positive macrophages significantly enhanced CD8^+^ T-cell proliferation, cytotoxicity, and cytokine production, which was associated with a better clinical prognosis [12]. Furthermore, interferons (IFNs) were shown to stimulate the polarization of Siglec-1-positive macrophages with T-cell-activating and tumor-inhibiting potential both in vitro and in vivo, and a PD-L1 blocking antibody further enhanced the antitumor effects of IFN-α [65]. However, in mice with triple-negative breast cancer, breast cancer cells were shown to promote PD-L1 expression in Siglec-1-positive macrophages by activating JAK2 signaling and promoting tumor immune escape. The infiltration of CD8^+^ T cells into the microenvironments of breast tumors and lung metastatic nodes was enhanced after the clearance of Siglec-1-positive macrophages, and the growth of in situ tumors and lung metastasis was inhibited [16]. Therefore, further studies are needed to elucidate the complex roles and mechanism of Siglec-1-positive macrophages in different TMEs and the signaling pathways and key cytokines involved in their regulation to clarify the role of macrophages in different tumors [18].

The outcomes of the interactions between Siglec-9 and its ligands depend on the stage of tumor growth and its microenvironment, as the immune response can be inhibited during early tumorigenesis, and antitumor immunity can be promoted once tumors are established. After tumor formation, Siglec-E deficiency enhances the differentiation of TAMs toward tumor-promoting M2-type macrophages and promotes tumor growth, and macrophage clearance reverses the effect of Siglec-E deficiency in mice, possibly because Siglec-E ligands can directly inhibit the formation of protumorigenic M2 macrophages and recode them into an antitumor phenotype. Thus, Siglec-9 might play dual roles in cancer progression [66]. However, a study [67] demonstrated that human Siglec-7/9 and Siglec-E inhibit the endogenous antitumor immune response as well as responses to tumor-targeting and immune checkpoint-inhibiting antibodies in vivo. They also restrict responses to tumor-targeting and checkpoint-targeting antibodies, thus demonstrating an advantage in combined immunotherapy. When MUC1 is expressed on cancer cells, it is decorated by multiple short, sialylated O-linked glycans (MUC1-ST), which bind to Siglec-9 to induce macrophages to display a TAM-like phenotype with increased expression of PD-L1 [47]. Similarly, Rodriguez et al. [68] showed that the increased expression and secretion of α-2,3 Sia in the TMEs of patients with PDAC promoted Siglec-9 receptor activation by upregulating CD206 and PD-L1 and immunosuppressive factors such as IL-10 and IL-6 monocyte polarization and differentiation to produce immunosuppressive TAMs, thereby promoting tumor progression and metastasis. Another study showed an upregulation of Siglec-9 on tumor-infiltrating T cells from patients with NSCLC, CRC, and ovarian cancer. Siglec-9–expressing T cells coexpressed several inhibitory receptors, including PD-1 [53]. In conclusion, the impact of these Siglecs on tumor progression is highly dependent on the anatomical distribution of the tumor and the local TME, and Siglec-7/9 blockade can significantly reduce the tumor burden in vivo, supporting the use of antibodies targeting Siglec-7/9 to therapeutically enhance antitumor immunity.

The expression of Siglec-10 on TAMs from ovarian and breast cancer patients can be specifically combined with CD24 expressed on tumor cells. Blocking CD24 or Siglec-10 with monoclonal antibodies can enhance the ability of macrophages to phagocytose tumor cells and slow tumor growth. When CD24 binds to Siglec-10, Siglec-10 triggers a signaling cascade by recruiting and activating proteins containing the SHP-1 and SHP-2 structural domains, which phosphorylates the ITIM region and blocks Toll-like receptor (TLR)-mediated inflammatory responses. This negatively regulates intracellular signaling, inhibits phagocytosis by macrophages and promotes tumor immune escape [37]. Recent research suggested that Siglec-10^hi^ TAMs were associated with an unfavorable prognosis in patients with HCC, and numerous M2-like signaling pathways were shown to be significantly upregulated in Siglec-10^hi^ TAMs. Moreover, blocking Siglec-10 promoted the antitumor efficacy of the PD-1 inhibitor [69]. Therefore, blocking CD24 or Siglec-10 with monoclonal antibodies enhances the ability of macrophages to phagocytose tumor cells and inhibit tumor growth; this approach may be useful for patients who are unresponsive to meditators of the PD-1/PD-L1 pathway.

Human Siglec-15 expression is upregulated in human tumor cells and/or tumor-infiltrating macrophages/myeloid cells, whereas it is expressed at low levels in macrophages from normal tissues. Siglec-15 is induced by M-CSF and recognizes the sialyl-Tn (sTn) antigen, which is commonly expressed in human cancers. In a coculture of THP-1 macrophage-like cells overexpressing Siglec-15 with lung cancer cells overexpressing sTn synthase, M-CSF induced M2-like macrophages to express Siglec-15, which was not upregulated on M1-like macrophages, and produced TGF-β via the DAP12-Syk pathway, thereby promoting tumor metastasis[29]. In addition to sTn and related antigenic sequences, Siglec-15 also has a high affinity for sialylated glycans and presumably plays an important role in their signaling function [70].

Overall, TAMs can change the TME in the tumor initiation stage. During tumor progression, TAMs can enhance the migration and invasion of tumor cells and inhibit the antitumor immune response. Siglecs can affect the polarization of TAMs by secreting inhibitory cytokines and affect the phagocytosis of TAMs to regulate the TME and tumor progression. When tumors develop, the TME induces abnormal increases in the expression of PD-1 and PD-L1. Activation of the PD-1/PD-L1 pathway significantly inhibits the immune response of T cells, resulting in the immune escape of tumors. Many Siglec molecules, including Siglec 1, 9, 10, and 15, are associated with PD-1/PD-L1 expression and may function in tumors through different pathways. Studies have shown that a single-agent checkpoint blockade (anti-PD-L1/PD-1) is rarely effective in some subgroups [71]. Although the relationship between the expression of Siglecs and PD-L1 in the TME is not clear, anti-PD-1 and anti-Siglec therapies in combination might have improved efficacy.

#### Siglecs weaken the killing effect of tumor-associated neutrophils

**Fig. 4:**
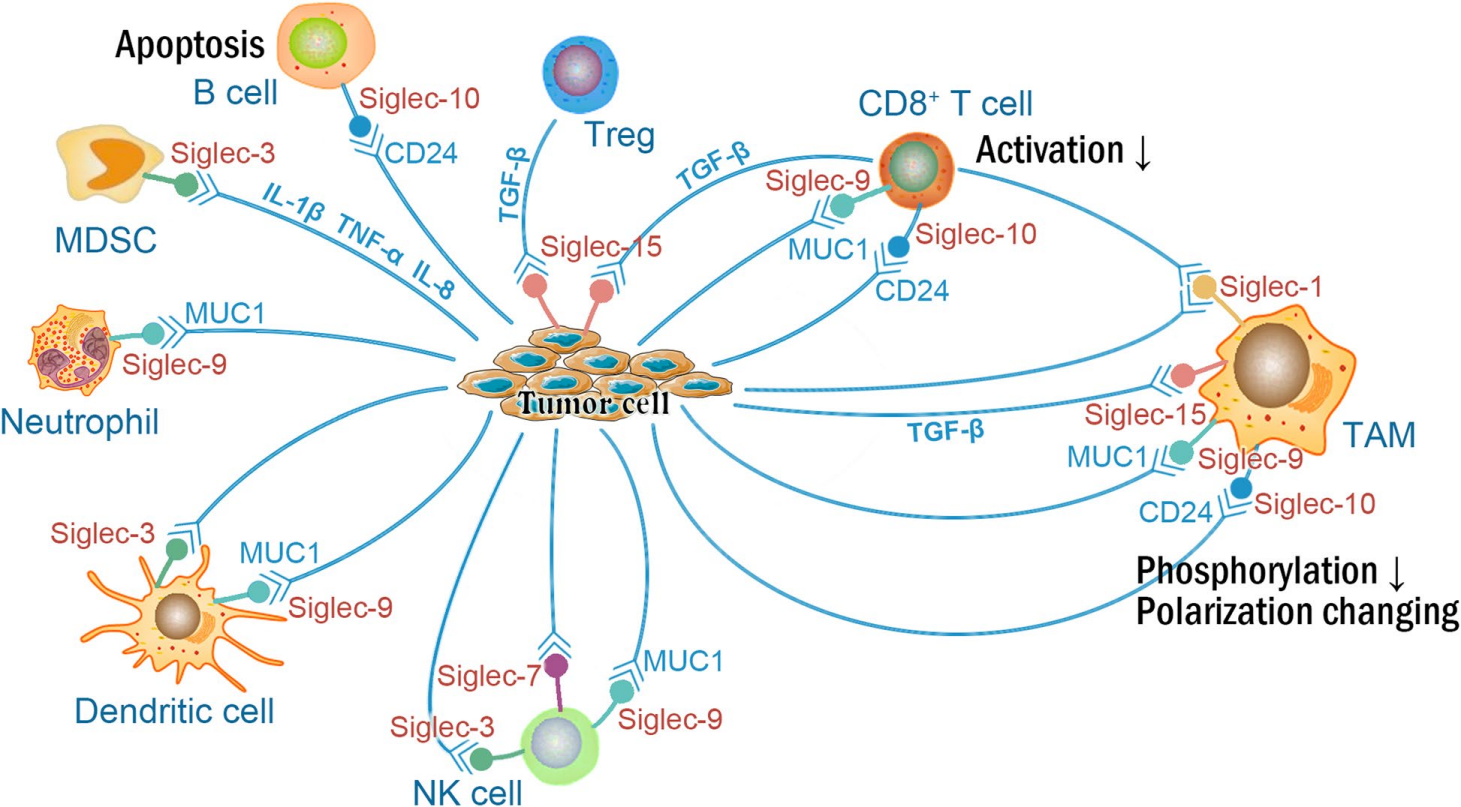
Schematic representation of possible functions of Siglec family members in regulation tumor biology

Siglec-9-positive neutrophils promote tumor immune escape. In vitro studies on CRC have shown that Siglec-9 induces SHP-1 recruitment and inhibits the neutrophil killing of tumor cells; moreover, the inhibitory effect is diminished after blockade with a Siglec-9 antibody [72]. In an in vivo experiment, neutrophils from Siglec-E-deficient mice showed an increased ability to kill tumor cells and enhanced immunosurveillance of autologous tumors [66]. Neutrophils play an important role in the occurrence and development of tumors. Siglecs can inhibit the killing effect of tumor-related neutrophils and affect tumor progression; therefore, analyses of the expression and functions of Siglec receptors in neutrophil subpopulations in the TME may provide more insight into tumor immunosuppression and immune escape. The possible roles of Siglec family members in regulating tumor biology are presented in Fig. 4.

## Targeting Siglecs in tumor therapy

Therapeutic targeting of the Sia-Siglec axis is promising for the treatment of tumors because Siglecs are mostly expressed in immune cells and affect the TME. Currently, monoclonal antibodies (mAbs) targeting Siglecs are applied to deplete tumor cells via passive immunotherapy [20]. Most mAbs specifically bind a target antigen and neutralize or stimulate its activity; however, newer therapeutic strategies, such as immune checkpoint inhibition, and T-cell engaging therapies, such as bispecific T-cell engaging (BiTE) single-chain antibody constructs and chimeric antigen receptor (CAR) T cells, have shown remarkable efficacy in clinical trials [73]. Here, we discuss drugs targeting Siglecs and their progress in their clinical application in tumor therapy (Table 2).

**Table 2:** Siglec-directed therapeutics for tumor

Siglec	Drug	Tumor type	Modality	Therapeutic strategy	Phase	Refs/Trial ID
Siglec-2 (CD22)	Epratuzumab	B cell lymphoma	MoAb	Inhibits the cis SA-Siglec-2 interaction for the induction of B cell signal transduction and caspase-dependent apoptosis	II	NCT00906841
	Inotuzumab ozogamicin	B cell lymphoma	MoAb	Causes cell death by inducing double-strand DNA breaks	I/II	NCT01363297 NCT01564784
	DT2219	Refractory B-lineage leukemia or lymphoma	BsAb	Inhibits the protein synthesis and apoptosis	I/II	NCT02370160
	CD22-CAR-T	Follicular lymphoma, ALL, NHL, Large cell lymphoma	CAR T	Recognises specific antibodies from tumour cells and targets them	I	NCT02315612
Siglec-3 (CD33)	lintuzumab	AML	MoAb	Induces cell death by complement and/or antibody-directed cellular cytotoxicity or as a direct effect of engaging the CD33 receptor	I/II	NCT03441048 NCT03867682
	Gentuzumab Ozogamicin	Newly diagnosed and relapsed AML	MoAb	Leads to cell death by causing site-specific, double-stranded breaks	II	NCT03374332
	AMG330	AML	BiTE	Directs cytotoxic T cells to CD33-expressed human AML cells killing the target	I	NCT02520427
	JNJ-67571244	Not responding AML patients at high risk of myelodysplastic syndrome	BiTE	Directs cytotoxic T cells to CD33-expressed human AML cells killing the target	I	NCT03915379
	CD33 CAR-T	AML	CAR T	T-cells are genetically changed to help target leukemia cells	I/II	NCT03126864
Siglec 6	Siglec-6 CAR-T	CLL	CAR T	Increases the activity of CARs that target membrane-distal epitopes	Not Applicable	[23]
Siglec 7	Ganglioside GD3	Melanoma	Vaccine	Modulate NK cell cytotoxicity	Not Applicable	NCT00597272
	Siglec-7 CAR-T	Solid Tumors	CAR T	Recognizes and eliminates tumor cells, in a non‐ histocompatibility complex molecule restricted way	Not Applicable	[74]
Siglec 9	Gatipotuzumab	Solid Tumors	MoAb	Activats the immune system to induce ADCC agains tumor cells	I/II	NCT01222624 NCT03360734
	Siglec-9 CAR-T	Solid Tumors	CAR T	Recognizes and eliminates tumor cells, in a non‐ histocompatibility complex molecule restricted way	Not Applicable	[74]
Siglec 10	Alemtuzumab	CLL	MoAb	Kills of tumour cells by CDC and ADCC	II	NCT01465334
Siglec 15	NC318	Advanced or metastatic solid tumors	MoAb	Restores normal T-cell function by blocking Siglec-15-mediated immunosuppression	I/II	NCT03665285

*AML* Acute myeloid leukemia, *ALL* Acute lymphoblastic leukemia, *NHL* Non-Hodgkin’s lymphoma, *CLL* Chronic lymphocytic leukemia, *CART* Chimeric antigen receptor-T cells, *MoAb*: Monoclonal antibody, *BsAb* Bispecific monoclonal antibody

### *Siglec-2*

(CD22) is a cell surface receptor expressed mostly on B cells that regulates B-cell proliferation, survival, signaling, and antibody production [27]. CD22 is an attractive therapeutic target considering its unique presence in B lymphocytes. Epratuzumab is a humanized IgG antibody against CD22 that phosphorylates CD22, affects BCR signaling by Ig crosslinking, and induces B-cell signal transduction and caspase-dependent apoptosis. Epratuzumab has been investigated in combination with chemotherapy or rituximab in both non-Hodgkin's lymphomas (NHLs) and B-cell acute lymphoblastic leukemia (B-ALL) [75]. Inotuzumab ozogamicin is an antibody–drug conjugate (ADC) comprised of a human anti-CD22 antibody attached to calicheamicin that utilizes the CD22 epitope for the targeted delivery of toxic payloads to B-cell lymphoma/leukemia cells. Currently, some clinical trials on other drugs in combination are ongoing [76]. DT2219 is a bispecific ligand-directed toxin incorporating single-chain variable fragments targeting CD19 and CD22 and is currently in early clinical trials [77]. Additionally, many ongoing trials evaluating CD22-directed chimeric antigen receptors (CARs), particularly in children with relapsed or refractory B-cell leukemia, are showing safety and efficacy [78]. Some novel CARs, such as bispecific CD20/CD22 CARs and CD19/CD22 CARs, are also in development [75].

#### Siglec-3

Siglec-3 is found on cells of myeloid lineage and AML progenitor cells but not on normal stem cells. It is a specific and ideal target for AML therapeutics. Gentuzumab ozogamicin was the first developed targeted agent and provided an overall survival benefit for a subset of patients but increased the AML mortality rate [79]. Another mAb, lintuzumab, an unconjugated antibody that induces cell death by an antibody-dependent cellular cytotoxicity (ADCC) mechanism or directly affects the CD33 receptor, has been studied in a series of clinical trials [80]. AMG 330 is a human bispecific T-cell engaging (BiTE) antibody directed against CD33/CD3 that leads to T-cell expansion, and a phase 1 trial is underway (NCT02520427) [81]. Anti-CD33 antibody-linked CAR-T cells showed effectiveness in targeting the AML cell line CD33. CAR-cytokine-induced killer (CIK) cells also showed significant antileukemic activity in vitro and are likely to enter early clinical trials [82].

#### Siglec-6

Siglec-6 is broadly expressed in CLL and warrants investigation as a candidate target for antibody-based immunotherapeutic interventions [83]. Recent research generated a fully human-derived anti-Siglec-6 CAR and showed that it effectively eliminated CLL cells in vitro and in xenograft models [23]. In conclusion, Siglec-6 is a possible target for CLL immunotherapy.

#### Siglec-7/9

Siglec-7/9 are recognized as inhibitory receptors, and they can promote immune suppression when bound to ligands. A recent study suggested that Siglec-7 CAR and Siglec-9 CAR can mediate antitumor activity in vitro against several tumor lines and, more importantly, in a xenograft mouse model of human tumors [74]. Novel antibodies that target Siglec-9 have been developed and have been shown to reduce the tumor volume in ovarian cancer [84]. The utilization of nanoparticles as a therapeutic delivery strategy has also been investigated, revealing the suppression of melanoma tumor growth in mice [85]. Targeted strategies for Siglec7/9 need to be further researched and clinically developed.

#### Siglec-10

Siglec-10 on the surface of immune cells can promote the immune escape of tumor cells by binding CD24 [28]. Alemtuzumab is a humanized monoclonal antibody that targets Siglec-10, which is expressed at high levels on the surface of B and T lymphocytes [86]. Clinical studies of alemtuzumab in combination with other drugs for the treatment of CLL are currently underway.

#### Siglec-15

Siglec-15 is a novel immunomodulatory target whose expression is significantly increased in a variety of tumor cells, such as lung, ovarian and head and neck cancer cells. NC318 is a mAb targeting Siglec-15 that blocks Siglec-15-mediated immune suppression and restores T-cell function in vitro, thereby exerting antitumor immune effects [87]. A phase I/II study of NC318 for head and neck cancer (HNSCC) and triple-negative breast cancer is underway and may be useful for anti-PD-1/PD-L1-resistant patients.

## Conclusion

The roles of the Siglec family are still being explored. In addition to promoting immune escape in tumors, Siglecs play roles in osteoporosis, infectious diseases, and allergic reactions. In other words, the interaction of Siglecs and their ligands may have differential functional results that are dependent on the background provided by the inhibition or activation of the members of the Siglec family, the immune cell subsets they express, the tumor type, the species, and other activation or inhibition signals in the TME. Targeted tumor immunotherapies targeting the Siglec family, such as some specific antibodies and artificial glycochain analogs, are gradually being applied. Factors in the TME can regulate the expression of sialiclycans in cancer cells and the expression of Siglecs in immune cell subsets. Therefore, before considering the use of Siglec blockade in immunotherapy, it is necessary to further study the different functions of Siglecs based on their expression in various cell types and the mechanisms by which they promote tumor immune escape to reduce the adverse reactions of tumor drugs. This strategy can also be combined with other therapies to exert a synergistic effect, thereby killing more tumor cells and treating tumors more effectively.

## Data Availability

The materials that support the conclusion of this review have been included within the article.

## Abbreviations

TME: Tumor microenvironment
DCs: Dendritic cells
NK cells: Natural killer cells
Sia: Sialic acid
DAMPs: Damage associated molecular patterns
ITAM: Immunoreceptor tyrosine-based activation motif
ITIM: Immunoreceptor tyrosine-based inhibition motif
SHPs: SH2 domain-containing protein tyrosine phosphatases
AML: Acute myelocytic leukemia
ALL: Acute lymphoblastic leukemia
CLL: Chronic lymphocytic leukemia
TAMs: Tumor-associated macrophages
PDAC: Pancreatic ductal adenocarcinoma
TGF-β: Transforming growth factor-β
OS: Osteosarcoma
MAPK: Mitogen-activated protein kinase
CTL: Cytotoxic T lymphocyte
NSCLC: Non-small cell lung cancer
RCC: Renal cell carcinoma
HCC: Hepatocellular carcinoma
TLR: Toll-like receptor
sTn: Sialyl-Tn
mAbs: Monoclonal antibodies
BiTE: Bispecific T-cell engaging
CAR: Chimeric antigen receptor
ADC: Antibody–drug conjugates
HNSCC: Head and neck cancer
